# Welcome to *Laboratory Animal Research*

**DOI:** 10.1186/s42826-019-0005-1

**Published:** 2019-06-24

**Authors:** Je Kyung Seong

**Affiliations:** Seoul National University, Seoul, South Korea

It is a great honour to introduce Springer’s new peer-reviewed journal, *Laboratory Animal Research* (*LAR*), which aims to provide a leading global forum in the broader area of animal sciences and experimental medicines.

Animal experimental research is crucial to making significant progress in medical-scientific research, in order to gain a better and more comprehensive understanding of healthcare; to develop new medications to prevent, diagnose, and cure diseases; and to investigate interactions between disciplines, including animal medicine, biology, pathology, genetics, and relevant sciences. Corresponding to the growing importance of animal experimental research, *LAR* is able to shed light on the discovery of various disease mechanisms through in vivo studies with model organisms, including genetics, behaviour, disease models, bioinformatics, and omics such as phenomics, genomics, and metabolomics.

The Korean Association for Laboratory Animal Science (KALAS) was established in 1985, to facilitate a discussion about animal genetics, the development of veterinary medicine, animal welfare, and sciences through quality research papers on the biology, physiology, anatomy, toxicology, pathology, and genetics of laboratory animals, animal models, animal behaviour and diseases, animal biotechnology, and other relevant topics**.** *LAR*, which has been organized by KALAS as a quarterly publication since 1985, purposes to disseminate and communicate up-to-date research findings with international readers, and to provide beneficial effects to the biomedical community. *LAR* seeks to publish high-quality research on laboratory animal research, to be the premiere academic journal in academia, and to lead the way in defining scholarship.

It is essential for the journal to have a good balance of different article types. *LAR* encompasses not only evidence-based empirical research articles and applied components which can have great impact on animal research, but also informed and thoughtful opinions on case reports. KALAS aspires to create an insightful, high-quality, and vibrant scientific journal in English with the widest readership possible. Although *LAR* began accepting English manuscripts in 2011, it has still had a limited reach to international readers due to its platform. Springer Nature, a highly-renowned and leading publisher of robust and insightful research around the world, is a proper partner for this professional and specialized publication’s mission to extend the reach of its papers.

I, on behalf of the *LAR* staff, must give special thanks to our editorial team for their enormous support of this effort. The editorial team has invited in eminent experts on a variety of specialties, and strives to make the journal a superb contribution to the discipline of animal research. *LAR* welcomes and appreciates your submissions of original research, whether individual or collaborative. The Associate Editors and I make a solid promise to minimize the time for review and decision-making. We look forward to seeing your work in *LAR*, an innovative venue for multi-disciplinary scholarly works.

